# Accuracy in use of adrenalin auto-injectors in a simulated emergency situation: a comparison of JEXT, EpiPen and Emerade

**DOI:** 10.1186/2045-7022-5-S3-O5

**Published:** 2015-03-30

**Authors:** Rebecca Knibb, Kirsty Morton

**Affiliations:** 1 Aston University, Birmingham, UK

## 

Correct use of an adrenalin auto-injector (AAI) is vital in an emergency situation where a person has gone into anaphylactic shock. Studies have shown that nearly half of untrained participants are unable to correctly use an AAI training device after instruction. Emerade is an AAI with a different design for use, with pictures providing instructions. This study aimed to assess intuitiveness and accuracy of use of JEXT, EpiPen and Emerade in untrained, non-allergic participants, in a simulated emergency situation.

Participants (n=90 adults) were randomly assigned to JEXT, EpiPen or Emerade. A simulated scenario involved a live patient acting unconscious after eating something they were allergic to; a loud ambulance siren played throughout. Participants were asked to give the person an injection of adrenalin in the leg, using a trainer pen with no instructions available. They were then asked to give a second shot with a pen of the same design with instructions. The simulation was scored by the researcher and video recorded; participants were interviewed about their experience.

No participant using EpiPen or JEXT successfully gave their patient adrenalin when they had no instructions to go by, compared to 82% using Emerade (p<0.001). After reading instructions, significantly more participants successfully gave their patient adrenalin using Emerade (100%) compared to JEXT (64%) or EpiPen (33%), p<0.001. Participants also took significantly less time to administer adrenalin with Emerade (mean=14.73 seconds), compared to JEXT (29.21) or EpiPen (33.72), p<0.001. Instructions on JEXT and EpiPen were confusing and skim read by participants, thus they missed important information. Emerade was reported to be easy to use both with and without instructions and pictures were easy to follow.

Emerade is an intuitive easy to use AAI compared to JEXT or EpiPen. In this simulated emergency situation participants found it difficult to read and act on written instructions. This is likely to be more pronounced in a real emergency where an AAI might be used by someone with little or no training. Instructions on AAIs need to be simplified with less complicated designs.

